# Papillomavirus infection in rural women in southern India

**DOI:** 10.1038/sj.bjc.6602348

**Published:** 2005-01-25

**Authors:** S Franceschi, R Rajkumar, P J F Snijders, A Arslan, C Mahé, M Plummer, R Sankaranarayanan, J Cherian, C J L M Meijer, E Weiderpass

**Affiliations:** 1 International Agency for Research on Cancer, 150 cours Albert Thomas, 69372 Lyon Cedex 08, France; 2 Christian Fellowship Community Health Centre, Ambilikai, Dindigul District, Tamil Nadu 624612, India; 3 Vrije University Medical Center, Postbus 7057, 1007 MB Amsterdam, The Netherlands; 4 Finnish Cancer Registry, Liisankatu 21B, 00170 Helsinki, Finland; 5 Karolinska Institutet, PO Box 281/Nobels väg 12A, 17 177 Stockholm, Sweden; 6 The Cancer Registry of Oslo, Montebello, 0310 Oslo, Norway

**Keywords:** human papillomavirus, cervical neoplasias, India

## Abstract

To investigate the prevalence of, and the risk factors for, cervical infection with 44 types of human papillomavirus (HPV) in a rural area in the Dindigul District, Tamil Nadu, India, we interviewed and obtained cervical cell samples from 1891 married women aged 16–59 years. HPV prevalence was 16.9% overall and 14.0% among women without cervical abnormalities, or 17.7 and 15.2%, respectively, age-standardised to the world standard population. In all, 21.9% of infections involved more than one HPV type. High-risk HPV types predominated, particularly HPV 16 (22.5% of women infected), followed by HPV 56, HPV 31, HPV 33 and HPV 18. Unlike most populations studied in developed countries, HPV prevalence was constant across the age groups. HPV positivity was inversely associated with education level (odds ratio (OR) among women with high school *vs* no education=0.6) and positively associated with widowhood and divorce (OR=1.7), nulligravidity (OR=2.3), and condom use (OR=2.6). It is unclear how much low clearance of, or frequent reinfection with HPV accounted for the study prevalence of infection in different age groups.

The prevalence of human papillomavirus (HPV) is low in some populations in Asia (Anh *et al*, 2003) and Europe (de Sanjosé *et al*, 2003), although it exceeds 15% in women aged 15–74 years in some parts of Latin America (Molano *et al*, 2002) and sub-Saharan Africa (Thomas *et al*, 2004), where incidence rates for cervical cancer are very high (Ferlay *et al*, 2001). HPV prevalence shows different patterns of infection by age in different populations. Much higher HPV prevalences were generally found below age 25 (Molano *et al*, 2002; Shin *et al*, 2003; Peto *et al*, 2004) or 35 years (Ronco *et al*, 2005) than above these ages, but in a very high HPV-prevalence area (Ibadan, Nigeria), the proportion of HPV-positive women was similar in all 10-year age groups between 15 and 65 years or more (Thomas *et al*, 2004).

Information on HPV prevalence and type-specific distribution among Indian women is scanty (Duttagupta *et al*, 2004). We have, therefore, carried out an HPV survey in a rural area in southern India.

## MATERIALS AND METHODS

We carried out the present study between February and October 2003 in the Dindigul District, Tamil Nadu, India as a collaborative project between the Christian Fellowship Community Health Centre (CFCHC) and the International Agency for Research on Cancer (IARC). The study area is rural, at an average elevation of 600 m above sea level in the foothills of the Western Ghats and consists of 324 village panchayats (local administrative structures consisting of 4–23 villages). The study goal was to assess the prevalence of cervical HPV infection and cytological abnormalities in a sample of 2000 married, nonpregnant women aged 16–59 years, but we searched for an approximately two-fold larger population to compensate for ineligibility and refusals. We therefore randomly selected a few villages in the study district, as follows.

After excluding the 113 village panchayats that participated in a concurrent IARC randomised trial of cervical visual screening (Sankaranarayanan *et al*, 2004), we stratified the remaining village panchayats according to their total population (small: <3000; intermediate: 3000–5000; large: ⩾5000 people). We then randomly extracted nine small size, three intermediate size, and one-third of a large size village panchayat in order to obtain an approximately equal number of women from each stratum. The study working team, consisting of one medical doctor (R Rajkumar), three *ad hoc* trained nurses, and three healthcare workers, stayed in each selected village for 2–5 days and visited each household to invite eligible women. Women who agreed to participate signed informed consent forms as recommended by the ethics committees of the CFCHC and IARC, both of which approved this study. A structured questionnaire including information on sociodemographic characteristics, reproductive factors and use of contraceptive methods was administered in the local language (Tamil) in women's homes and an appointment was arranged in the temporary clinic that had been installed in each village during the survey.

### Gynaecological examination and specimen collection

Women underwent a pelvic examination, performed by one of the three nurses. None had undergone a hysterectomy. Samples of exfoliated cells from the ecto- and endocervix were collected with a broom-type brush and placed in vials of PreservCyt solution, according to the manufacturer's instructions (Cytyc, Boxborough, MA, USA). All samples were stored at +4°C until shipment to the Department of Pathology at the Vrije University Medical Center, Amsterdam, The Netherlands, for HPV testing and liquid-based cytology. In order to meet the immediate screening needs of participating women, visual inspection with acetic acid and, in the presence of suspicious lesions, colposcopy, were performed (Sankaranarayanan *et al*, 2004). Acetowhite lesions were treated, mainly with cryotherapy. Women with cervicovaginitis received antibiotic treatment. When cytology results became available, women with high-grade squamous intraepithelial lesions (HSIL) were recalled, biopsied and treated with cryotherapy or loop electrosurgical excision as appropriate.

### Cytology

Liquid-based ThinPrep cytology slides were prepared from PreservCyt vial specimens according to the manufacturer's standard protocol. Results were classified by cytotechnicians according to the CISOE-A classification (Bulk *et al*, 2004), and abnormal smears were reviewed by an experienced cytopathologist. Cytological evidence of infection with *Trichomonas (T.) vaginalis* was reported systematically.

### Human papillomavirus detection techniques

To analyse the quality of the target DNA for polymerase chain reaction (PCR) testing, cervical specimens were screened with beta (*β*)-globin gene-specific primers. HPV positivity was assessed by general primer-mediated GP5+/GP6+-PCR and by hybridisation of PCR products in an enzyme immunoassay (EIA) using two HPV oligoprobe cocktails that, together, detect the following 44 HPV types: HPV 6, 11, 16, 18, 26, 30, 31, 32, 33, 34, 35, 39, 40, 42, 43, 44, 45, 51, 52, 53, 54, 55, 56, 57, 58, 59, 61, 64, 66, 67, 68, 69, 70, 71 (equivalent to CP8061), 72, 73, 81 (equivalent to CP8304), 82 (IS39 and MM4 subtypes), 83 (equivalent to MM7), 84 (equivalent to MM8), cand85, 86, cand89 (equivalent to CP6108) and JC9710. PCR products that were positive in the EIA were subsequently subjected to further typing by reverse line blot hybridisation, as described previously (van den Brule *et al*, 2002).

High-risk HPV types for this analysis included HPV 16, 18, 26, 31, 33, 35, 39, 45, 51, 52, 53, 56, 58, 59, 66, 68, 73 and 82 (Muñoz *et al*, 2003). The group of low-risk types included all other HPV types.

### Statistical analysis

Odds ratios (ORs) for HPV positivity with 95% confidence intervals (CIs) by various women's characteristics were calculated using unconditional, multiple logistic regression adjusted for age group (<25, 25–34, 35–44, 45–54, ⩾55 years). The statistical significance of trends for ORs was assessed by giving increasing scores to successive categories of appropriate variables in the logistic model.

## RESULTS

Of the 2884 women invited, 154 could not be enrolled in the study because they were pregnant, 31 because of illness and 13 because of heavy menstrual bleeding, thus leaving 2686 eligible candidates. There were 743 refusals (27.7%) mainly because of lack of time (372) and reluctance to undergo a gynaecological examination (331). Women who refused to participate were similar to the participating ones in relation to their occupation, husband's occupation, religion, marital status, age at marriage, number of pregnancies and use of contraceptive methods. The only characteristics that were associated with refusals were age (highest below age 25 and above age 54) and education (with the minority of women with high school or college having a participation 10% lower than less educated women).

**Figure 1:**
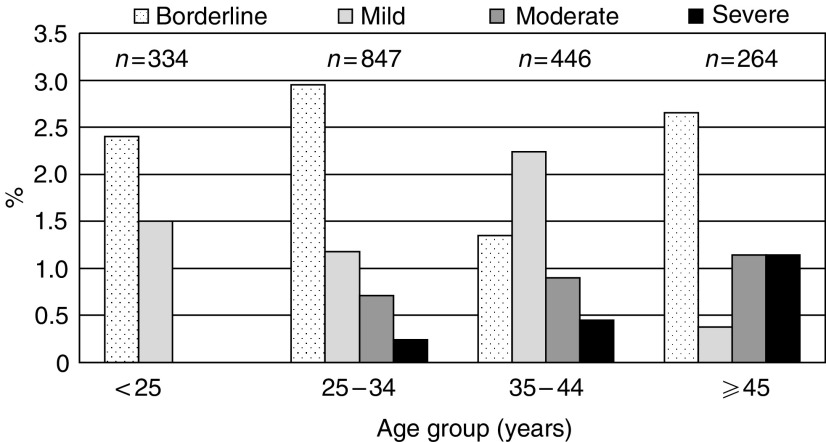
Prevalence of dyskaryosis at liquid-based cytology by degree of dyskaryosis and by age group. (Dindigul, India).

Of the 1943 women who underwent a pelvic examination, 62 women had acetowhite lesions on visual inspection, 49 had inadequate cytology results and three had *β*-globin-negative HPV samples. Of the 1891 women with valid cytology and HPV results, 92 (4.9%) had abnormal cytological findings, including 46 with borderline dyskaryosis, 26 with mild dyskaryosis, 13 with moderate dyskaryosis and seven with severe dyskaryosis. Whereas the frequency of borderline dyskaryosis was similar in different age groups, moderate or severe dyskaryosis was never found in women under age 25, and steadily increased from age 25–34 (1.0%) to age 45–59 (2.3%, Figure 1). None of the study women reported previous cytological screening for cervical cancer.

The prevalence of HPV of any type was 16.9%, but it varied between 14.0% among cytologically normal women and 73.9% among those with cytological abnormalities (Table 1). The corresponding proportions age-standardised to the world population were 17.7% overall, 15.2% among those without and 64.9% among those with cytological abnormalities. In total, 250 women had single-type and 70 had multiple-type infection.

**Figure 2:**
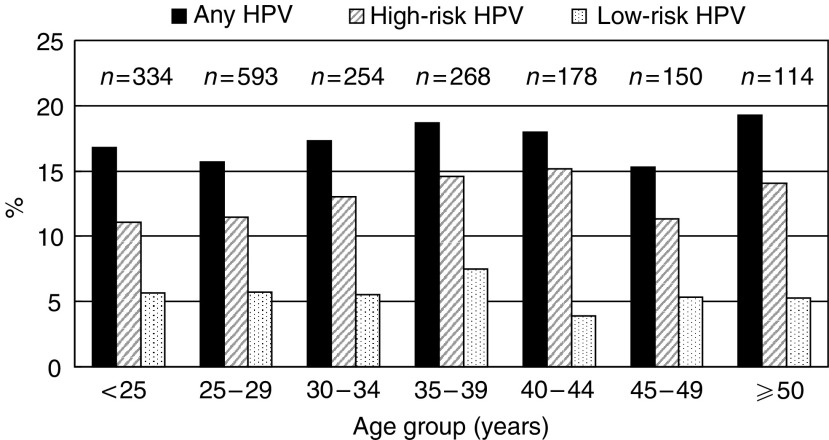
Age-specific prevalence of cervical human papillomavirus (HPV) DNA. (Dindigul, India).

High-risk HPV types were substantially more frequent (12.5% of all women) than low-risk types (6.0%). Most commonly found in either single- or multiple-type infections were HPV 16 (3.8%), HPV 42 (a low-risk type, 2.2%), HPV 56 (1.5%), HPV 31 (1.2%), HPV 33 (1.2%) and HPV 18 (1.0%), but HPV type distribution varied by cytological results. High-risk types were found in 70.7% of women with cytological abnormalities and in 17 out of 20 (85.0%) women with moderate or severe dyskaryosis (including HPV 16 or 18 in nine women). Details of HPV types found in women with multiple HPV infections are given in Appendix A (Table A1). The prevalence of HPV (any type, high- and low-risk types, separately) was similar in women in different age groups (Figure 2, Table 2).

Tables 2 and 3 show the relationship between HPV positivity and major characteristics of study women after adjustment for age. Education level was inversely associated with HPV prevalence (OR for women with high school or college degrees *vs* illiterate women=0.6; 95% CI: 0.4–1.0), whereas women working outside the house (the vast majority as farm labourers) had an excess of HPV positivity of borderline statistical significance (OR *vs* housewives=1.3; 95% CI: 1.0–1.6, Table 2). Husband's occupation and religion, which was Hinduism in the majority of study women, were not associated with HPV positivity (Table 2).

Women who were widowed (OR=1.7; 95% CI: 1.0–2.7), and separated or divorced (OR=2.3; 95% CI: 0.8–6.7) showed a higher proportion of HPV positivity than married women (Table 3). The median age at marriage was 18 years (range=12–33) and the median number of pregnancies was three (range=0–14). The median age at first pregnancy was 19 years (range=13–34). Child deliveries after age 30 were reported by only 5.9% of women. Age at first marriage and number of pregnancies among ever-pregnant women were unrelated to HPV positivity, although an OR of 2.3 (95% CI: 1.3–4.2) was found among the few nulligravidae (3% of all study women). Spontaneous or voluntary abortions were reported by 21.6% of HPV-negative and 25.9% of HPV-positive women (data not shown). Among contraceptive methods, only tubal ligation was reported often (63% of study women) and was unrelated to HPV positivity. An increased risk of HPV positivity was found, however, among 30 women who reported condom use (OR=2.6; 95% CI: 1.2–5.7). Finally, cytological evidence of infection with *T. vaginalis* was found in 6.0% of study women and was associated with an OR for HPV positivity of 1.5 (95% CI: 0.9–2.3, Table 3).

When education level, marital status, number of pregnancies, and contraceptive methods were included, in addition to age, in the same multiple logistic regression, the corresponding ORs did not change materially.

## DISCUSSION

The main findings of our large survey in Dindigul District in rural India are the high overall prevalence of HPV infection, the similar prevalence across different age groups, and the marked predominance of HPV 16 among HPV types.

All-age HPV prevalence in Dindigul District is similar to that found in high-risk areas for cervical cancer in Latin America (Molano *et al*, 2002; Matos *et al*, 2003), although lower than in some parts of sub-Saharan Africa (Thomas *et al*, 2004). No clear peak in HPV prevalence was found in young women in Dindigul District, whereas in most countries studied so far the prevalence of HPV below age 25 is ⩾2-fold higher than at 45 or above (Molano *et al*, 2002; Peto *et al*, 2004). However, a constant prevalence of HPV across age groups has been recently reported in Nigeria (Thomas *et al*, 2004), suggesting that in some poor countries the risk of HPV infection is similar in different generations of women in contrast to the markedly higher HPV prevalence among younger women in many developed countries (Peto *et al*, 2004). Low rates of HPV clearance and frequent HPV reinfections may also contribute to the steady age pattern observed in Dindigul District and Nigeria (Thomas *et al*, 2004).

India shows some of the highest rates of cervical cancer worldwide (Ferlay *et al*, 2001), particularly in rural areas (Rajkumar *et al*, 2000). The combined prevalence in a rural area of West Bengal of HPV types 16 and 18 in 534 Hindu women and 478 Muslim women was 7.5 and 9.6%, respectively (Duttagupta *et al*, 2004). HPV 16 and 18 positivity was similar in four age groups (<25, 25–34, 35–44, ⩾45 years) among Muslim women, but steeply declined with age among Hindu women.

Our survey is the first study to assess a broad range of HPV types in different age groups in India in a large sample of women. It showed that HPV 16 is the most frequently found type in the general population, affecting 22.5% of HPV-positive women. The corresponding percentages in IARC surveys in Asia and Africa were less than 14% (Shin *et al*, 2003; Thomas *et al*, 2004). The predominance of HPV 16 in the Indian population is similar to that found with the same PCR-assay in Europe (32.6%, Ronco *et al*, 2005) and Latin America (22.0%, Molano *et al*, 2002). Of note, a high proportion (63.2%) of cervical cancer specimens was also found to be positive for HPV 16 in India (Franceschi *et al*, 2003).

Among other HPV types, a relatively high frequency of HPV 42 and HPV 56 (the second most frequent high-risk type in Dindigul) was found in previous IARC surveys (Anh *et al*, 2003; Thomas *et al*, 2004). HPV 42 is a low-risk type while HPV 56 seems to be one of the high-risk types like HPV 31, 33, 35, 52, 53 and 58 that is substantially under-represented in invasive cancer compared to preinvasive cervical lesions (Clifford *et al*, 2003).

A strength of our survey in Dindigul District is a reliance on high-quality liquid-based cytology in a population that had never been screened before. Most previous surveys in developing countries (Duttagupta *et al*, 2004), including those by IARC, had to rely on locally read cytology and indeed showed a much lower agreement between cytological findings and HPV results than in our present study, where 85% of moderate and severe dyskaryosis (corresponding to high-grade squamous intraepithelial lesions, HSIL) harboured high-risk HPV types. Moderate or severe dyskaryoses were never found below 25 years in Dindigul District, but their prevalence increased with age and in women aged 45 or older it was approximately four-fold greater than in women in the same age group in England (Department of Health, 2000).

Only a few of the possible correlates of HPV positivity could be evaluated, since inclusion of unmarried women and questions about sexual habits were discouraged during our preparatory meetings with the village heads and local collaborators in Dindigul. The study took place, however, in a rather homogenous, low-income rural population and it is, therefore, not surprising that the very few characteristics that stood out as being directly (e.g., nulligravidity, widowhood or divorce, and condom use) or indirectly (high school or college degree) associated with HPV positivity involved a small, and sometimes very small, proportion of study women.

The uniformity of living standards and lifestyle in rural India provides some reassurance in respect of selection bias. Two main groups of women accounted for nonparticipation in our study: those who were not traced during the few days that the gynaecological clinic stayed in each village (approximately 25% of women), and those who were contacted but refused to participate (an additional 19%) mainly out of a lack of familiarity with gynaecological examinations. However, we were able to obtain some basic information from almost all women during the survey, or in subsequent repeated visits to the same villages. The three main groups (participants, refusals, and untraced women) were similarly distributed by sociodemographic and reproductive factors.

## Figures and Tables

**Table 1:** Prevalence of various human papillomavirus (HPV) types by cytological findings and overall among 1891 women (Dindigul, India)

	**Cytology**								
	**Normal**			**Abnormal**			**Total**		
**HPV type^a^**	**Single**	**Multiple**	**Total (%)**	**Single**	**Multiple**	**Total (%)**	**Single**	**Multiple**	**Total (%)**
Negative			1547 (85.9)			24^b^ (26.1)			1571 (83.1)
Positive, any	199	53	252 (14.0)	51	17	68 (73.9)	250	70	320 (16.9)
High risk	125	47	172 (9.6)	49	16	65 (70.7)	174	63	237 (12.5)
Low risk	68	34	102 (5.7)	2	9	11 (12.0)	70	43	113 (6.0)
Uncharacterised	6	––	6 (0.3)	––	––	0	6	––	6 (0.3)
									
*High risk*									
16	30	21	51 (2.8)	15^c^	6^d^	21 (22.8)	45	27	72 (3.8)
18	8	7	15 (0.8)	3^e^	1	4 (4.3)	11	8	19 (1.0)
26	1	0	1 (0.1)	0	0	0	1	0	1 (0.1)
31	11	3	14 (0.8)	7^d^	2^e^	9 (9.8)	18	5	23 (1.2)
33	8	6	14 (0.8)	4	4^d^	8 (8.7)	12	10	22 (1.2)
35	7	7	14 (0.8)	1	3^d^	4 (4.4)	8	10	18 (1.0)
39	4	6	10 (0.6)	0	1^d^	1 (1.1)	4	7	11 (0.6)
45	3	3	6 (0.3)	2^d^	1	3 (3.3)	5	4	9 (0.5)
51	5	3	8 (0.4)	5^e^	1	6 (6.5)	10	4	14 (0.7)
52	11	2	13 (0.7)	1	0	1 (1.1)	12	2	14 (0.7)
53	1	2	3 (0.2)	0	1	1 (1.1)	1	3	4 (0.2)
56	14	5	19 (1.1)	7^d^	3	10 (10.9)	21	8	29 (1.5)
58	1	2	3 (0.2)	3	1^d^	4 (4.4)	4	3	7 (0.4)
59	11	1	12 (0.7)	1	0	1 (1.1)	12	1	13 (0.7)
66	6	3	9 (0.5)	0	0	0	6	3	9 (0.5)
73	2	1	3 (0.2)	0	0	0	2	1	3 (0.2)
82	2	2	4 (0.2)	0	0	0	2	2	4 (0.2)
									
*Low risk*									
6	2	2	4 (0.2)	0	1	1 (1.1)	2	3	5 (0.3)
30	3	0	3 (0.2)	0	1	1 (1.1)	3	1	4 (0.2)
32	1	2	3 (0.2)	0	0	0	1	2	3 (0.2)
40	2	4	6 (0.3)	0	0	0	2	4	6 (0.3)
42	25	12	37 (2.1)	1	3	4 (4.4)	26	15	41 (2.2)
43	0	3	3 (0.2)	0	2	2 (2.2)	0	5	5 (0.3)
44	0	1	1 (0.1)	0	0	0	0	1	1 (0.1)
54	3	2	5 (0.3)	0	0	0	3	2	5 (0.3)
55	1	1	2 (0.1)	0	0	0	1	1	2 (0.1)
67	1	2	3 (0.2)	0	2	2 (2.2)	1	4	5 (0.3)
69	1	0	1 (0.1)	0	0	0	1	0	1 (0.1)
70	3	3	6 (0.3)	0	0	0	3	3	6 (0.3)
81	5	3	8 (0.4)	0	2	2 (2.2)	5	5	10 (0.5)
83	1	0	1 (0.1)	0	0	0	1	0	1 (0.1)
84	1	1	2 (0.1)	0	0	0	1	1	2 (0.1)
85	5	0	5 (0.3)	0	0	0	5	0	5 (0.3)
86	2	0	2 (0.1)	0	0	0	2	0	2 (0.1)
CP6108	3	0	3 (0.2)	0	0	0	3	0	3 (0.2)
JC9710	9	9	18 (1.0)	1	0	1 (1.1)	10	9	19 (1.0)

a The same woman can appear more than once in ‘Multiple’ and ‘Total’ columns.

b Includes three high-grade squamous intraepithelial lesions.

c Includes six high-grade squamous intraepithelial lesions.

d Includes one high-grade squamous intraepithelial lesion.

e Includes two high-grade squamous intraepithelial lesions.

**Table 2:** Detection of cervical human papillomavirus (HPV) DNA according to sociodemographic characteristics among 1891 women^a^ (Dindigul, India)

		**HPV DNA positive**			
	**Number of women**	**Number**	**(%)**	**OR^b^**	**(95% CI)**
*Age (years)*					
<25^c^	334	56	(16.8)	1	
25–34	847	137	(16.2)	0.96	(0.68–1.35)
35–44	446	82	(18.4)	1.12	(0.77–1.63)
45–54	228	39	(17.1)	1.02	(0.65–1.60)
⩾55	36	6	(16.7)	0.99	(0.39–2.50)
*χ*^2^_1_ for trend				0.23	*P*=0.63
					
*Education*					
Illiterate^c^	869	158	(18.2)	1	
Primary or secondary	754	128	(17.0)	0.91	(0.70–1.19)
High school or college	261	32	(12.3)	0.63	(0.41–0.95)
*χ*^2^_1_ for trend				4.00	*P*=0.05
					
*Occupation*					
Manual worker/farmer	1022	189	(18.5)	1.28	(0.99–1.64)
Housewife^c^	812	122	(15.0)	1	
Others	53	8	(15.1)	0.99	(0.46–2.18)
					
*Husband's occupation*					
Manual worker^c^	1132	192	(17.0)	1	
Farmer	431	68	(15.8)	0.91	(0.67–1.23)
Others	172	24	(14.0)	0.79	(0.50–1.25)
					
*Religion*					
Hindu^c^	1643	284	(17.3)	1	
Christian	240	36	(15.0)	0.83	(0.57–1.21)
Muslim	6	0	(0.0)	0.00	(0.00–∞)

a Figures do not add up to the total because of missing values.

b Adjusted for age.

c Reference category; OR=Odds ratio; CI=Confidence interval.

**Table 3:** Detection of cervical human papillomavirus (HPV) DNA according to marital status, reproductive factors, contraceptive use and *Trichomonas vaginalis* infection among 1891 women^a^ (Dindigul, India)

		**HPV DNA positive**			
	**Number of women**	**Number**	**(%)**	**OR^b^**	**(95% CI)**
*Age at marriage (years)*					
⩽15^c^	351	59	(16.8)	1	
16–17	528	96	(18.2)	1.10	(0.77–1.57)
18–19	566	91	(16.1)	0.95	(0.66–1.36)
⩾20	438	69	(15.8)	0.92	(0.63–1.35)
*χ*^2^_1_ for trend				0.57	*P*=0.45
					
*Marital status*					
Married^c^	1767	288	(16.3)	1	
Widowed	107	26	(24.3)	1.66	(1.03–2.67)
Separated or divorced	16	5	(31.3)	2.30	(0.79–6.71)
					
*Age at menarche (years)*					
⩽12^c^	332	58	(17.5)	1	
13–14	887	156	(17.6)	1.01	(0.72–1.41)
⩾15	628	91	(14.5)	0.79	(0.55–1.14)
*χ*^2^_1_ for trend				2.15	*P*=0.14
					
*Number of pregnancies*					
0	59	17	(28.8)	2.31	(1.26–4.22)
1–2^c^	848	128	(15.1)	1	
3–4	749	137	(18.3)	1.22	(0.92–1.62)
⩾5	235	38	(16.2)	1.03	(0.66–1.60)
*χ*^2^_1_ for trend				0.09	*P*=0.76
					
*Contraceptive method*					
None^c^	657	118	(18.0)	1	
Tubal ligation	1183	189	(16.0)	0.85	(0.65–1.11)
Condom	30	11	(36.7)	2.63	(1.22–5.68)
Others^d^	13	1	(7.7)	0.38	(0.05–2.95)
					
*Trichomonas vaginalis infection*					
Negative^c^	1777	294	(16.5)	1	
Positive	114	26	(22.8)	1.48	(0.94–2.34)

a Figures do not add up to the total because of missing values.

b Adjusted for age.

c Reference category.

d Including seven users of oral contraceptives and six users of intrauterine device; OR=Odds ratio; CI=confidence interval.

**Table A1:** Combinations of human papillomavirus (HPV) types in 70 women with multiple infections (Dindigul, India)

**HPV type**	**No.**	**HPV type**	**No.**	**HPV type**	**No.**
6,**16**	1	**16**,JC9710	1	**35,66**	1
6,**18**	1^a^	**18,35**	1	**35**,70	1
6,**56**	1	**18**,40	1	**35**,81	1^a^
**16,18**	2	**18,53**,42	1	**39,56**	1
**16,18,39**	2	30,81	1^a^	40,43	1
**16,33**	2^a^	**31,33**	1^a^	40,54,**59**	1
**16,33,39**	1	**31,39**	1^a^	42,43	1
**16,35**,40	1	**31**,42	1	42,**51**	2^a^
**16,39**	2	**31,51**	1	42,**52**	1
**16**,42	2	**31,82**	1	42,**53**	1
**16**,42,43	1^a^	32,JC9710	2	42,81,JC9710	1
**16,51**,55,**66**,JC9710	1	**33,45**	2	42,**82**	1
**16,53**	1^a^	**33,52**	1	43,**45**	2^a^
**16,56**	3^a^	**33,56**	2^b^	54,**58**	1
**16**,67	3^b^	**33**,JC9710	1	**58**,84,JC9710	1
**16**,67,81	1	**35**,42	2^a^	**66**,JC9710	1
**16**,70	1	**35**,42,**56**	1	70,JC9710	1
**16,73**	1	**35**,44	1		
**16**,81	1	**35,58**	1^a^		

High-risk types in bold face.

a One woman with abnormal cervical findings.

b Two women with abnormal cervical findings.

## Appendix A

Table A1 shows the details of HPV types found in women with multiple HPV infections.
